# Tissue Distribution of the Readthrough Isoform of AQP4 Reveals a Dual Role of AQP4ex Limited to CNS

**DOI:** 10.3390/ijms21041531

**Published:** 2020-02-24

**Authors:** Claudia Palazzo, Pasqua Abbrescia, Onofrio Valente, Grazia Paola Nicchia, Shervin Banitalebi, Mahmood Amiry-Moghaddam, Maria Trojano, Antonio Frigeri

**Affiliations:** 1 Department of Basic Medical Sciences, Neurosciences and Sense Organs, School of Medicine, University of Bari Aldo Moro, 70124 Bari, Italy; clapalazzo@hotmail.it (C.P.); onofrio.valente@uniba.it (O.V.); maria.trojano@uniba.it (M.T.); 2 Department of Bioscience, Biotechnology and Biopharmaceutics, University of Bari Aldo Moro, 70125 Bari, Italy; graziapaola.nicchia@uniba.it; 3 Division of Anatomy, Department of Molecular Medicine, Institute of Basic Medical Sciences, University of Oslo, 0317 Oslo, Norway; shervin.banitalebi@studmed.uio.no (S.B.); mahmo@medisin.uio.no (M.A.-M.)

**Keywords:** aquaporin-4, AQP4ex, translational readthrough, astrocyte endfeet, membrane localization, translational recoding

## Abstract

Translational readthrough (TRT) of aquaporin-4 (AQP4) has remarkably expanded the importance of this new post-transcriptional mechanism, as well as the regulation potential of AQP4. The TRT isoform of AQP4, named AQP4ex, is central for both AQP4 polarization and water channel activity in the central nervous system (CNS). Here we evaluate the relevance of the TRT mechanism by analyzing whether AQP4ex is also expressed in peripheral tissues and whether the expression of AQP4ex is necessary for its polarized expression as it occurs in perivascular astrocyte processes. To this purpose, AQP4ex null mice were used, and analysis was performed by immunolocalization and immunoblot. The results demonstrate that AQP4ex is expressed in kidney, stomach, trachea and skeletal muscle with the same localization pattern as the canonical AQP4 isoforms. AQP4ex protein levels vary from 6% to about 13% of the total AQP4 protein levels in peripheral tissues. Immunogold electron microscopy experiments demonstrated the localization of AQP4ex at the astrocytic endfeet, and experiments conducted on AQP4ex null mice CNS confirmed that the expression of AQP4ex is necessary for anchoring of the perivascular AQP4. Without the readthrough isoform, AQP4 assemblies are mis-localized, being uniformly distributed on the astrocyte processes facing the neuropile. No alteration of AQP4 polarization was found in AQP4ex null kidney, stomach, trachea or skeletal muscle, suggesting that AQP4ex does not have a role for proper membrane localization of AQP4 in peripheral tissues. We conclude that a dual role for AQP4ex is limited to the CNS.

## 1. Introduction

Aquaporins (AQPs) are a family of membrane water transporting proteins that facilitate water movement across the cell plasma membrane in the presence of hydrostatic and/or osmotic gradients [1]. Among the AQPs, aquaporin-4 (AQP4) is the central nervous system (CNS) water channel mainly expressed in perivascular foot processes of astrocytes and in ependymal cells delimiting the ventricles [2,3].

An interesting feature of AQP4 is its peculiar protein expression, influenced by the 5’UTR sequence, as two isoforms: a full-length, long (M1) isoform with translation initiation at Met-1 of 32 kDa and a shorter (M23) isoform produced by alternative splicing with translation initiation at Met-23 of 30 kDa [4,5]. AQPs generally form homotetramers in the cell plasma membrane, whereas in the case of AQP4, the M1- and M23 isoforms form heterotetramers [6], which further aggregate in the plasma membrane to form well-ordered two-dimensional supramolecular structures, called orthogonal arrays of particles (OAPs), observable by freeze fracture electron microscopy (FFEM) [7,8]. Both AQP4 isoforms function as water channels, although their relative abundance is tissue-specific and affects binding of pathogenic neuromyelitis optica antibodies [9].

AQP4 may be subjected to two types of regulation: short-term regulation via phosphorylation [10,11] and long-term regulation by transcriptional and post-transcriptional control, which influence expression, function and assembly in OAPs [6,12,13]. Recently, a new AQP4 isoform (i.e., AQP4ex) produced by a translational readthrough mechanism has been identified [14]. AQP4ex contains a C-terminal extension of 29 amino acids from the canonical stop codon to a downstream more efficient stop codon [15]. All four isoforms are involved to define OAP formation and size: M23 forms large OAPs, while the N-terminus (M1) and the C-terminus extensions contribute to hone OAP sizing in transfected cells [14]. Moreover, phosphorylation of residues Ser331 and Ser335 of the extended isoforms seems to play an important role in the short-term regulation of channel gating and water permeability [14]. Recently, to evaluate the functional role of AQP4ex we generated a transgenic mouse model in which AQP4ex is completely abolished using the CRISPR-Cas9 technique [16]. AQP4ex-KO mice revealed that AQP4ex is essential for the localization of AQP4 at the perivascular astrocytic membrane domains, even if large OAPs made of M1 and M23 canonical isoforms are still expressed. More interestingly, AQP4ex is also necessary to generate the NMO-IgG epitope in mice.

AQP4 is also expressed in many peripheral tissues, including collecting duct principal cells in kidney, gastric parietal cells and fast-twitch fibers of skeletal muscle [17,18,19,20]. Moreover, AQP4 is expressed in sensory organs, including retinal Muller cells, supporting cells in olfactory epithelium and in the organ of Corti in the inner ear [21].

In the present study, we evaluated to what extent is the readthrough mechanism involved in AQP4ex expression. We assessed whether AQP4ex is only expressed in the CNS or in all tissue where canonical AQP4 is expressed. Furthermore, we investigated whether the expression of AQP4ex in extra CNS tissues affects polarized expression as it occurs in the perivascular astrocytes.

## 2. Results

As a first step, to assess if the tissue distribution of the AQP4ex isoform overlaps with that of the canonical AQP4 isoforms (i.e., M1 and M23), its expression in the stomach, kidney, trachea, skeletal muscle and central nervous system (CNS) of mice was investigated by immunofluorescence. In addition, immunoblotting experiments were also performed using lysate samples from several tissues in which AQP4 has been found expressed. Moreover, experiments were performed in parallel on AQP4ex-KO samples in order to investigate the effect of AQP4ex suppression on the expression and the localization of the canonical isoforms.

**Figure 1:**
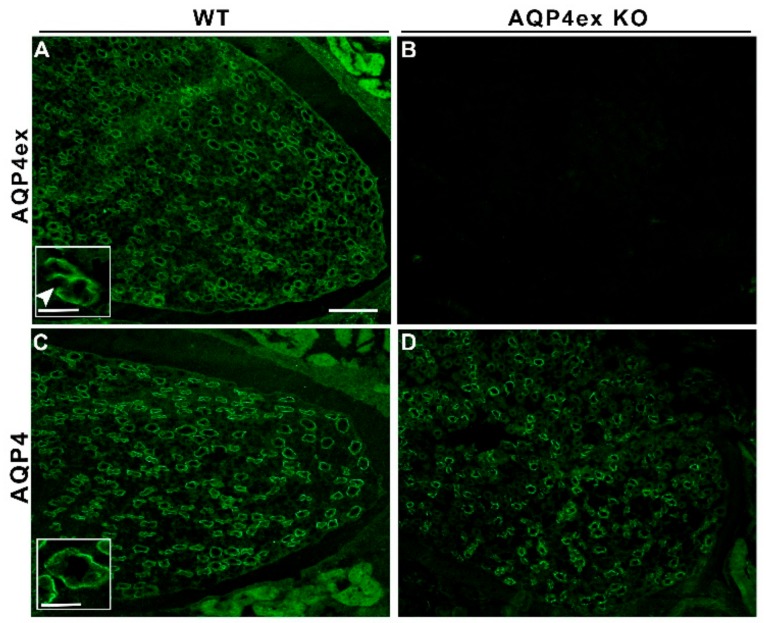
Immunolocalization of AQP4ex isoform in the kidney. Low-magnification of renal medulla stained with AQP4ex polyclonal antibodies (**A**,**B**) and AQP4 global antibodies (**C**,**D**). AQP4ex is strongly expressed in kidney-collecting duct cells (**A**), while the signal is absent in AQP4ex-KO tissue (**B**). Note that in AQP4ex-KO mice, the canonical AQP4 is expressed and correctly localized (**D**). Inserts showing the staining of the basolateral membrane of principal cells with AQP4ex (**A**) and AQP4 (**C**) antibodies on WT samples at higher magnification, arrowhead indicates unstained intercalated cell. Scale bars: 100 μm and 25 μm (insert).

As shown in Figure 1, immunofluorescence analysis showed a strong AQP4ex staining in the kidney-collecting duct in WT tissue (Figure 1A). AQP4ex staining was restricted to the basolateral membrane of principal cells in the collecting duct (Figure 1A, insert), while intercalated cells were not stained, as previously described for AQP4 [17,22]. The specificity of the AQP4ex staining was confirmed by the use of AQP4ex-KO mice, in which no renal expression of AQP4ex was detected. Interestingly, the suppression of AQP4ex did not affect the expression or localization of the canonical isoforms, indicating that the membrane domain expression of AQP4 is not dependent on AQP4ex.

**Figure 2:**
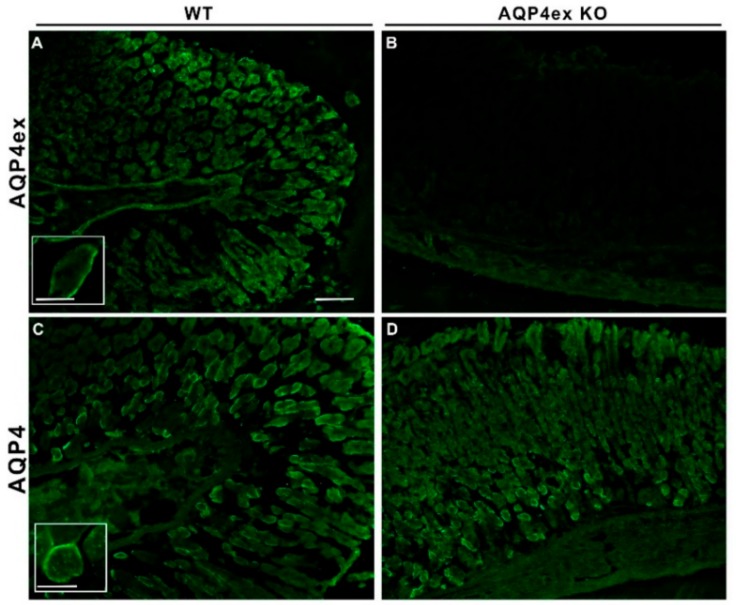
Immunolocalization of AQP4ex isoform in the stomach. At low magnification, AQP4ex is expressed in gastric glands of wild-type (WT) stomach (**A**) similar to that obtained with global antibodies (**C**). The signal is absent in AQP4ex-KO mouse (**B**). Inserts showing the staining localized at the basolateral membrane of parietal cells at higher magnification. Note that canonical isoforms appear correctly expressed in AQP4ex-KO tissue (**D**). Scale bars: 100 μm and 25 μm (inserts).

Immunofluorescence experiments were also performed to localize AQP4ex in different regions of mouse stomach mucosa (Figure 2). AQP4ex was consistently localized at the basolateral membranes of parietal cells along the axis of the gastric glands. This expression did not differ from that of the canonical isoforms [2,23,24]. AQP4ex basolateral expression of parietal cells was completely abolished in AQP4ex-KO mice (Figure 2B), indicating the specificity of the staining limited to the acid-secreting cells. Immunofluorescence analysis of the canonical AQP4 isoforms in AQP4ex-KO stomachs did not show an appreciable alteration of the staining, suggesting, as found in kidneys, that AQP4ex is not essential for the AQP4 membrane domain localization.

**Figure 3:**
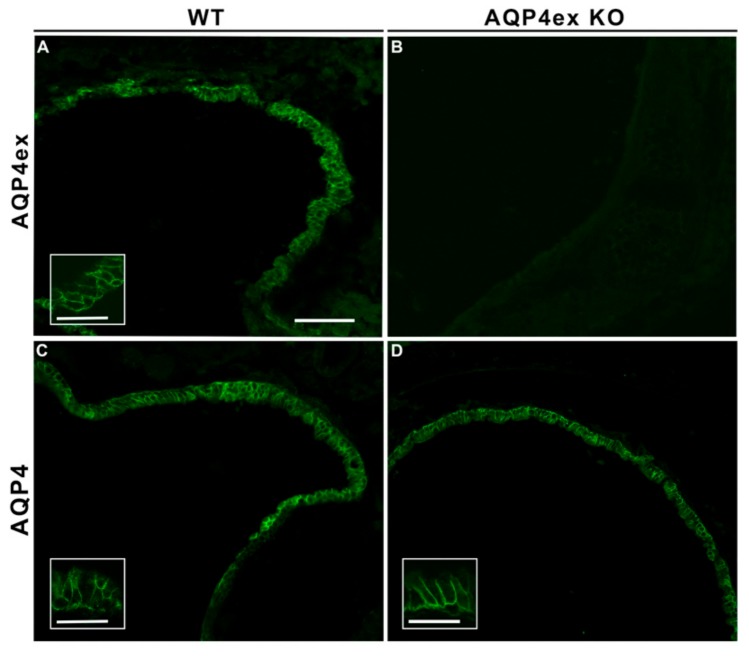
Immunolocalization of AQP4ex isoform in trachea. At low magnification, AQP4ex is expressed in the tracheal epithelium of wild-type (WT) mice (**A**) similar to that obtained with global antibodies (**C**). The signal is absent in AQP4ex-KO mice (**B**). Inserts showing the staining localized at the basolateral membrane of epithelial cells at higher magnification. Canonical isoforms appear normally expressed in AQP4ex-KO mice (**D**). Scale bars 50 μm, 25 μm (inserts).

In trachea, AQP4ex localized to the basolateral membrane of epithelial cells (Figure 3). The specificity of the AQP4ex staining was confirmed by the immunofluorescence on AQP4ex-KO mice, in which, as expected, no AQP4ex expression was detected. In wild-type tissues, canonical AQP4 colocalized with the extended isoforms and the suppression of AQP4ex did not affect the localization of the canonical isoforms in the correct cellular domain, indicating that the membrane expression of AQP4 is not dependent on AQP4ex, as in other peripheral tissues examined in the present study.

**Figure 4:**
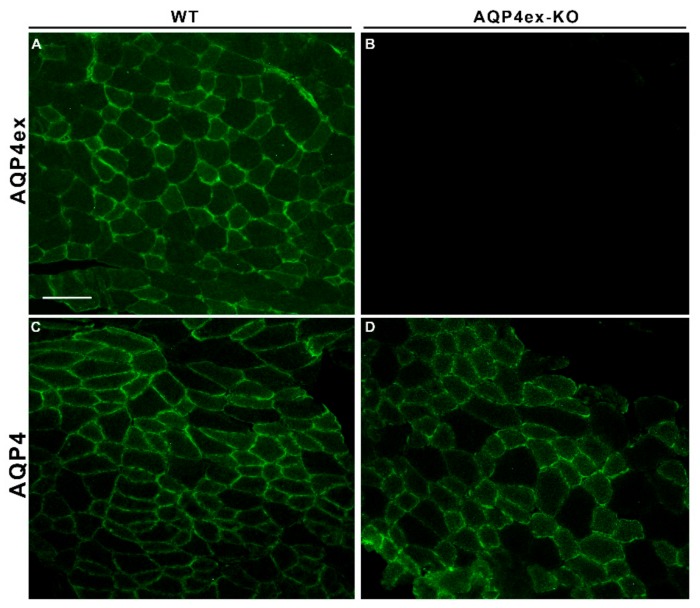
Expression of AQP4ex in skeletal muscle. Immunolocalization of AQP4ex in tibialis anterior of wild-type (WT) and AQP4ex-KO mice. Note the mosaic pattern of AQP4ex (**A**), similar to that of canonical AQP4 (**C**), indicative of staining of the sarcolemma of fast fibers. AQP4ex staining is totally abolished in AQP4ex-KO muscle (**B**). Canonical isoforms appear normally expressed in AQP4ex-KO mice (**D**). Scale bar 100 µm.

Expression of AQP4ex was also evaluated in skeletal muscle (Figure 4). Immunofluorescence signal was detected with a similar pattern to the canonical AQP4 [25], indicating that AQP4ex is expressed at the sarcolemma of fast-twitch fibers. No evident alteration was detected of the AQP4 residual protein in AQP4ex-KO skeletal muscle, suggesting that the expression of the extended form is not a determining factor for AQP4 sarcolemma expression.

**Figure 5:**
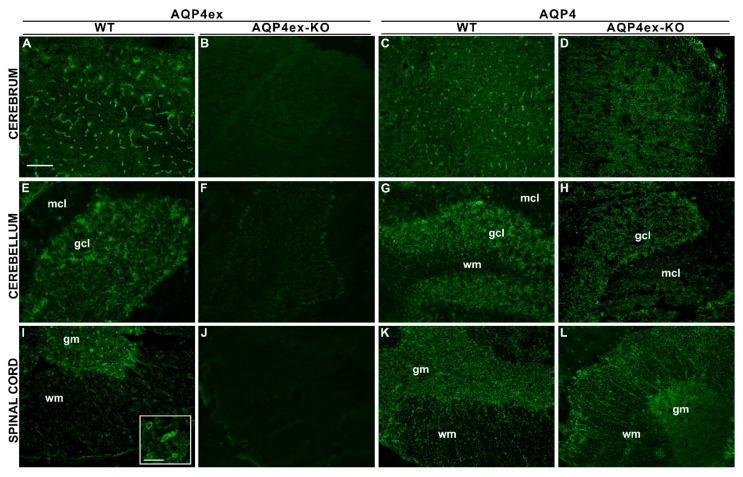
Immunolocalization of AQP4ex in the CNS. Wild-type (WT) cerebral cortex and cerebellum (granular, gcl, and molecular cell layer, mcl) stained with AQP4ex antibody show a strong perivascular distribution of the AQP4ex isoform (**A**,**E**). AQP4ex in the WT spinal cord (**I**) is localized in the inner gray matter (gm), particularly in pericapillary astrocyte processes, but it is also observable in the external white matter (wm). The insert (panel I) shows a magnified view of the gm area with vessels strongly stained by AQP4ex in WT spinal cords. As expected, AQP4ex antibody immunoreactivity is abolished in all AQP4ex-KO tissues (**B**,**F**,**J**). Note that, in AQP4ex-KO tissues, the typical perivascular AQP4 expression found in WT sections (**C**,**G**,**K**) is lost (**D**,**H**,**L**). Scale bar: 100 μm and 30 μm.

We also evaluated the distribution of AQP4ex and AQP4 in the CNS, analyzing cerebrum, cerebellum and spinal cord (Figure 5). Most of the AQP4ex staining was confined, as recently reported [16], to the pericapillary astrocyte endfeet of the cerebral cortex (Figure 5A) and at the perivascular astrocyte processes in the granule cell layer (gcl), with an additional staining of the dense network of astrocyte processes of the cerebellum gcl (Figure 5E). In the spinal cord, AQP4ex was highly expressed in both white and gray matter (Figure 5I). In the white matter, AQP4ex was distributed in astrocyte processes with a radial pattern protruding from the gray matter to the glia limitans externa. In gray matter, AQP4ex displayed the classic perivascular staining deriving from astrocytes endfeet, surrounding capillaries, but significant staining was also observed in the diffuse network of astrocyte processes. As expected, the immunofluorescence signal of AQP4ex was completely abolished in AQP4ex-KO mice, demonstrating the specificity of the staining and confirming the perivascular localization of AQP4 extended isoforms observed in WT samples.

The global AQP4 antibody on AQP4ex-KO sections revealed that the perivascular AQP4 staining of the cerebral cortex, spinal cord and in the gcl was lost, while the staining of reticular processes appeared poorly affected (Figure 5H,L).

In the absence of the extended isoforms, the filamentous staining in the spinal cord white matter was still intensely observable; furthermore, the perivascular signal from AQP4ex observed in WT tissues was totally abolished. These data confirm that suppressing AQP4ex expression totally eliminates the perivascular staining of AQP4, confirming, as found in other CNS areas [16], that AQP4ex expression is fundamental for the expression of AQP4 at the perivascular interface.

**Figure 6:**
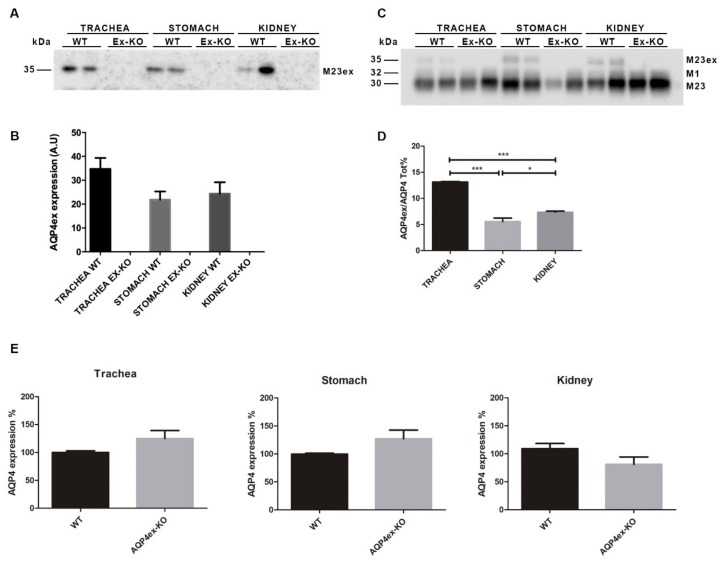
Immunoblot detection of AQP4 isoforms expressed in WT and AQP4ex-KO mice trachea, stomach and kidney. (**A**) A protein of 35 kDa was detected by anti-AQP4ex specific antibody in WT tissues and not in knockout (KO) tissues. (**B**) Densitometric analysis of the expression levels of AQP4ex in the selected tissues. (**C**) Protein extracts were also probed with the global anti-AQP4 antibody showing bands at 30, 32, and 35 corresponding to AQP4-M23, AQP4-M1 and AQP4-M23ex, respectively. (**D**) Densitometric analysis of the percentage of AQP4ex relative to the total AQP4 measured by immunoblotting (one-way ANOVA, * *p* < 0.05 and *** *p* < 0.0001, *n* = 3). (**E**) Bar charts showing the level of AQP4 expression (all isoforms) in WT and AQP4ex-KO tissues mean ± SE. (Student’s *t*-test, *n* = 3 for trachea and stomach and *n* = 5 for kidney).

Immunoblot experiments were performed (Figure 6) to evaluate quantitatively the expression levels of AQP4ex. A major band of ~35 kDa, corresponding to AQP4-M23ex, was detected in WT samples (Figure 6A) but not in the AQP4ex knockout mice extracts, as reported in the CNS [16]. The results were confirmed by probing the blot membranes with the commercial AQP4 antibody that recognizes all AQP4 isoforms. Densitometric analysis showed that the extended isoforms correspond to ~6% in stomach, ~7% kidney and ~13% trachea. These values are in the same range as those observed in the CNS, approximately 10% of all isoforms, with the highest expression in the cerebellum [16,26]. As previously observed [16], the amount of the canonical AQP4 isoform increased in the CNS of AQP4ex-KO mice. A similar tendency was observed in the peripheral tissues (Figure 6E).

**Figure 7:**
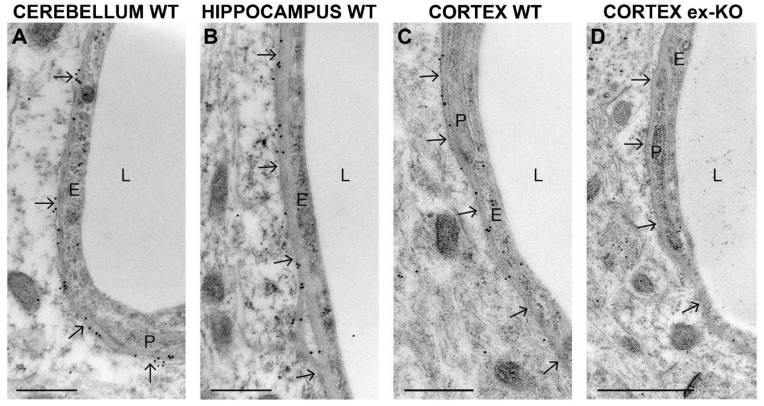
Subcellular localization of AQP4ex in astroglial cells. Portions of perivascular structures and cells from four CNS subregions are shown: cerebellum (**A**), hippocampus (**B**) and cerebral cortex from (**C**) WT and cerebral cortex from AQP4ex-KO (ex-KO) mice (**D**). The endothelium (E), the capillary lumen (L) and a pericyte (P) are also observable in the picture. Arrows indicate the perivascular astrocyte endfeet membrane. Immunogold particles can be seen along the perivascular astrocyte endfeet membrane of WT mice (arrows in A, B and C). Note that perivascular labeling of extended isoforms is abolished in the AQP4ex-KO cortex. Scale bars (A–C): 500 nm, (D): 1 um.

High-resolution immunogold cytochemistry was performed in order to evaluate in more detail the CNS expression of AQP4ex and the effect of its alteration in AQP4ex-KO mice. High density of gold particles was found in perivascular astrocyte processes in the cerebellum granular cell layer (Figure 7A), hippocampus (Figure 7B) and cerebral cortex (Figure 7C) of WT mice, confirming immunofluorescence data. The perivascular immunogold labeling was totally abolished in control sections from AQP4ex-KO mice (Figure 7D).

**Figure 8:**
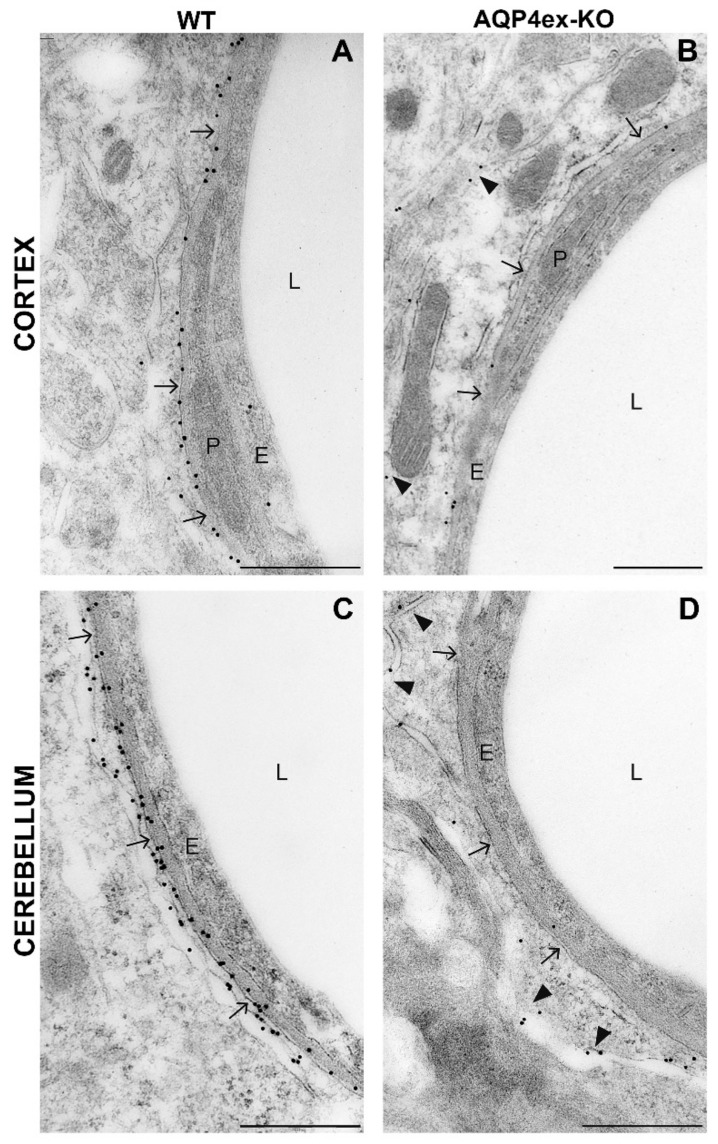
Immunogold labeling of AQP4 in the CNS of AQP4ex-KO mice. Representative images from WT (**A**,**C**) and AQP4ex-KO (**B**,**D**) mice cerebral cortex and cerebellum. Each picture shows endothelium (E), the capillary lumen (L) and a pericyte (P). Arrows indicate the astrocyte endfeet membrane domains facing the vessels, and arrowheads indicate astrocyte membranes facing the neuropile. The polarized AQP4 localization at the perivascular membrane domains facing the vessels observed in WT mice is lost in the AQP4ex-KO brain (arrows). Note, in AQP4ex-KO images the gold particles are located on the astrocyte membrane facing the neuropile (arrowheads, B and D) being indicative of mis-localization of AQP4. Scale bar: 500nm.

To evaluate the effect of the perivascular AQP4ex absence on the major AQP4 isoforms (M23 and M1) at the nanometric scale, immunogold staining was performed with anti-AQP4 antibodies detecting all AQP4 isoforms on AQP4ex-KO sections. The selective deletion of extended isoforms in AQP4ex-KO mice led to a dramatic reduction in perivascular AQP4 labeling in all CNS regions analyzed, as summarized by immunogold images from the cortex and cerebellum (Figure 8). Notably, images from AQP4ex-KO mice (Figure 8B,D) indicate a redistribution of canonical AQP4 from the perivascular membrane domains facing the vessel to the membrane domains facing the neuropile (arrowheads). This redistribution confirmed that AQP4ex is fundamental for AQP4 polarization and also that OAPs only consisting of canonical AQP4 isoforms are not retained at the perivascular pole, resulting in mis-localization.

## 3. Discussion

The recent discovery of AQP4ex as a translational readthrough isoform of AQP4 has remarkably expanded the importance of this new post-transcriptional mechanism, as well as the regulation capabilities of AQP4. The C-terminal extension of AQP4ex contains several putative functional domains. Two of these domains have been recently assessed: the phosphorylation site for the regulation of water transport activity and the targeting domain required for the interaction with the scaffolding protein α-syntrophin [14,16].

In the present study, we have investigated whether the readthrough is a generic mechanism or restricted specifically to the CNS. Immunolocalization and immunoblot experiments demonstrated that AQP4ex is also expressed in peripheral tissue with the same membrane localization as the canonical isoform. This indicates that the translational readthrough is a fundamental functional mechanism that permits the expression of AQP4ex in all the tissues where AQP4 is found. Furthermore, AQP4ex protein expression levels greatly differ from tissue to tissue, indicating that the TRT mechanism can be subjected to regulation, likely depending on the functional requirements of the AQP4ex isoform. These data promise an important physiological role for extended isoforms even outside the CNS and highlight the importance of the TRT mechanism in gene regulation.

Another interesting finding of the present study is that the tail of AQP4ex is not necessary to localize AQP4 in the specific membrane domain in peripheral tissues, as occurs in the CNS.

TRT has been suggested as a novel gene regulatory mechanism that allows a specific protein to have multiple targeting [27]. Examples of this multiple localization are the lactate and malate dehydrogenase enzymes whose peroxisome targeting is controlled by TRT [28,29]. In the case of AQP4, some similarities could be shown. Two independent targeting signals are present in AQP4: the first, in line with a study of Madrid and collaborators [30], located in the canonical AQP4 isoform is necessary for its basolateral membrane localization in epithelial cells; the second, specifically generated by TRT and, thus, localized in the C-terminal tail of AQP4ex, is required for anchoring at the perivascular astrocytic membrane domain. This second targeting signal may promote additional post-translational modification that could influence binding to other proteins or its tertiary/quaternary protein structure. In this regard, the presence of phosphorylation sites in the extended portion is suggestive of such an influence. Thus, AQP4ex is of dual functional significance in normal brain functions: firstly, as a water channel, and secondly, for anchoring AQP4 at the perivascular astrocyte endfeet, a membrane domain in which the absence of a tight junction (TJ) cannot limit lateral diffusion of membrane proteins. In the basolateral membranes of epithelial cells where lateral diffusion is TJ-limited, the functional role of AQP4ex appears not to be related to membrane localization. In this case, the principal function of AQP4ex should be the regulation of water flux at the basolateral membrane.

Hence, we can conclude that AQP4ex at the astrocyte endfeet has two important roles: spatial confinement and water transport regulation. In this dual role, proteins such as α-syntrophin [31,32,33] and dystrophin-associated proteins have a central role in anchoring AQP4 through AQP4ex [34,35,36]. In the basolateral membrane, the cytoskeletal proteins seem to have a minimal role, since the absence of AQP4ex does not affect AQP4 localization, probably due to the more secure role of the TJ. This also suggests that the interaction with cytoskeletal proteins appears to be of less importance compared to that with the CNS. However, it cannot be excluded that AQP4ex interaction with cytoskeletal proteins is still necessary for water channel activity in peripheral tissues. This possibility will be investigated in the future.

## 4. Materials and Methods

### 4.1. Animals

Experiments were conducted in accordance with the European directive on animal use for research, and the project was approved by the Institutional Committee on Animal Research and Ethics of the University of Bari and by the Italian Health Department (project license number 571/2018-PR and n° 2A298.N.2G1 of 27 July 2018). Mice were kept under a 12-h dark-to-light cycle, constant room temperature and humidity (22 ± 2 °C, 75%), with food and water ad libitum. Experiments were carried out on mice of different ages from 6 to 12 months old. Wild-type and AQP4ex-KO mice were anesthetized by intraperitoneal injection of ketamine (100 mg/kg body weight). All experiments were designed to minimize the number of animals used and their suffering.

### 4.2. Antibodies

The following primary antibodies were used: custom rabbit polyclonal anti-AQP4 (GenScript Biotech, Piscataway, NJ, USA) at a concentration of 0.4 μg/mL for immunoblot analysis and 0.7 μg/mL for immunofluorescence and custom rabbit polyclonal anti-mouse AQP4ex generated against the peptide DSTEGRRDSLDLASC within the mouse AQP4 carboxy-extension (GenScript Biotech, Piscataway, NJ, USA) at a concentration of 0.5 μg/mL for immunoblotting and immunofluorescence [16]. For immunofluorescence, AlexaFluor 488 anti-rabbit was used (Life Technologies, Thermo Fisher Scientific, Carlsbad, CA, USA) at a concentration of 1 μg/mL; for immunoblotting, anti-rabbit IgG-HRP (Bio-Rad, Hercules, CA, USA) was used following the manufacturer’s instructions.

### 4.3. Immunofluorescence on Tissue Sections

Immunofluorescence experiments were performed as previously described [37]. Briefly, tissues were fixed in 4% PFA solution at 4 °C overnight and, then, after washing for 1 h in PBS, were immersed in 30% sucrose solution in PBS overnight. After washing in PBS, tissues were embedded in Tissue-Tek OCT compound (Sakura, The Netherlands) and frozen at −80 °C. Sections of 8 μm thickness were cut on a cryostat (CM 1900; Leica, Wetzlar, Germany) at −20 °C and stored on poly-L-lysine glass slides (positively charged) at −80 °C. After blocking, sections were incubated with primary antibodies for 1 h at room temperature in blocking solution (0.1% gelatin in PBS), washed for 30 min and then incubated with secondary antibodies for 45 min. Finally, the sections were washed for 15 min in PBS and mounted in PBS-glycerol (1:1) pH 8.0 containing 1% n-propyl gallate. Finally, sections were viewed with an automated inverted Leica TCS SP8 confocal Lightning microscope using 20×/0.55, 40×/0.80 HC PL FLUOTAR and HC PL APO 63×/1.40 Oil CS2 objectives.

### 4.4. Sample Preparation for SDS-PAGE

For SDS-PAGE, the cerebral cortex from the left hemisphere, cervical portion of spinal cord, left cerebellum, kidney medulla and stomach were employed. Harvested tissues were frozen in liquid nitrogen and stored at −80 °C. For SNC tissues, proteins were extracted in 5–7 volumes of RIPA buffer (10 mM Tris-HCl, pH 7.4; 140 mM NaCl; 1% Triton X-100; 1% Na^+^deoxycholate; 0.1% SDS; 1 mM Na_3_VO_4_; 1 mM NaF and 1 mM EDTA) added with a cocktail of protease inhibitors (Roche, Milan, Italy). The lysis was performed on ice for 1 h, and the samples were then centrifuged at 21,000× *g* for 1 h at 4 °C. The supernatant was collected, and the proteins were measured with a bicinchoninic acid (BCA) Protein Assay Kit (Thermo Scientific, Waltham, MA, USA).

Peripheral tissues were mechanically homogenized in five volumes of ice-cold homogenizing buffer (HB: 250 mM sucrose; 10 mM Tris-HCl, pH 7.5) added with a protease inhibitor cocktail (Roche Diagnostics GmbH, Mannheim, Germany). The homogenates were centrifuged at 4000× *g* for 10 min at 4 °C and the supernatants centrifuged at 17,000× *g* for 45 min at 4 °C to obtain a membrane vesicle-enriched low-speed pellet [37]. These pellets were resuspended in five volumes of HB, and the protein content was measured as previously described.

### 4.5. SDS-PAGE and Western Blot Analysis

The electrophoresis and immunoblotting were performed as previously described [38]. Briefly, proteins were separated on 13% SDS/PAGE and transferred to polyvinylidenedifluoride (PVDF) membranes (Millipore, Burlington, MA, USA) for immunoblot analysis. Membranes were incubated with primary antibodies overnight, washed and incubated with peroxidase-conjugated secondary antibodies at room temperature for 45 min. Reactive proteins were revealed using an enhanced chemiluminescent detection system (Clarity Western ECL Substrate, Bio-Rad) and visualized on a Chemidoc Touch Imaging System (Bio-Rad). Densitometry analysis was performed using Image Lab software (Bio-Rad). The Band Analysis tool was used to select the bands, and a total protein normalization was performed, comparing bands of interest to the total protein content in each lane based on No-Stain™ Protein Labeling Reagent (Invitrogen, CA, California, USA). The amount of AQP4ex was measured on AQP4-probed immunoblots, calculating the percentage of AQP4ex (M23ex) relative to the average value of the total AQP4 (M23ex + M1 + M23) in each lane.

### 4.6. Perfusion and Tissue Preparation for Electron Microscopy

Adult WT and AQP4ex-KO mice (*n* = 3) were anesthetized and transcardially perfused with 4% paraformaldehyde and 0.1% glutaraldehyde in 0.1 M phosphate buffer (PB, NaPi) at a physiological pH of 7.4. After perfusion, the brains were removed, post-fixed overnight in the fixation solution and stored in a 1:10 dilution of the same fixative in 0.1 M PB.

### 4.7. Postembedding Immunogold Electron Microscopy

Brain sections were harvested and cut into 0.5–1mm tissue blocks. Hippocampus, parietal cortex and cerebellum were dissected, cryo-protected and quick-frozen in liquid propane, then subjected to freeze substitution and treated as previously described [39].

Immunogold cytochemistry staining were performed as previously described [39,40]. Briefly, ultrathin sections were incubated overnight with primary antibodies diluted in Tris-buffered saline with Triton X-100 (TBS-T) with 2% (*w/v*) human serum albumin (HAS) (Sigma-Aldrich, St. Louis, MI, USA) or 0.2% skimmed milk powder (for experiments using antibody to AQP4ex) at RT. Thereafter, the sections were rinsed and incubated with goat anti-rabbit (GAR) secondary antibody coupled to colloidal 15nm gold particles (Abcam, Cambridge, UK) diluted in TBS-T containing 2% (*w/v*) HSA and polyethylene glycol (PEG) (1:20). Finally, the sections were rinsed with dH_2_O, dried and incubated in 2% uranyl acetate followed by an incubation in 0.3% lead citrate. Immunostained sections were examined by a TECNAI 12 transmission electron microscope at 60kV (FEI, Hillsboro, OR, USA).

### 4.8. Statistical Analysis

Mean ± standard error is reported in the results. Statistical analysis was performed using GraphPad Prism 5 (GraphPad, San Diego, CA, USA) by *t*-test for unpaired data or analysis of variance (ANOVA), followed by Tukey’s test. A *p*-value < 0.05 was considered statistically significant.
